# Methodologic and Data-Analysis Triangulation in Case Studies: A Scoping Review

**DOI:** 10.1177/01939459241263011

**Published:** 2024-07-30

**Authors:** Margarithe Charlotte Schlunegger, Maya Zumstein-Shaha, Rebecca Palm

**Affiliations:** 1 Department of Health Professions, Applied Research & Development in Nursing, Bern University of Applied Sciences, Bern, Switzerland; 2 Faculty of Health, School of Nursing Science, Witten/Herdecke University, Witten, Germany; 3 Department of Health Care Research, Carl von Ossietzky University Oldenburg, Oldenburg, Germany

**Keywords:** case studies, nurse practitioners, primary health care, review, triangulation

## Abstract

**Aim:**

We sought to explore the processes of methodologic and data-analysis triangulation in case studies using the example of research on nurse practitioners in primary health care.

**Design and methods:**

We conducted a scoping review within Arksey and O’Malley’s methodological framework, considering studies that defined a case study design and used 2 or more data sources, published in English or German before August 2023.

**Data sources:**

The databases searched were MEDLINE and CINAHL, supplemented with hand searching of relevant nursing journals. We also examined the reference list of all the included studies.

**Results:**

In total, 63 reports were assessed for eligibility. Ultimately, we included 8 articles. Five studies described within-method triangulation, whereas 3 provided information on between/across-method triangulation. No study reported within-method triangulation of 2 or more quantitative data-collection procedures. The data-collection procedures were interviews, observation, documentation/documents, service records, and questionnaires/assessments. The data-analysis triangulation involved various qualitative and quantitative methods of analysis. Details about comparing or contrasting results from different qualitative and mixed-methods data were lacking.

**Conclusions:**

Various processes for methodologic and data-analysis triangulation are described in this scoping review but lack detail, thus hampering standardization in case study research, potentially affecting research traceability. Triangulation is complicated by terminological confusion. To advance case study research in nursing, authors should reflect critically on the processes of triangulation and employ existing tools, like a protocol or mixed-methods matrix, for transparent reporting. The only existing reporting guideline should be complemented with directions on methodologic and data-analysis triangulation.

Case study research is defined as “an empirical method that investigates a contemporary phenomenon (the ‘case’) in depth and within its real-world context, especially when the boundaries between phenomenon and context may not be clearly evident. A case study relies on multiple sources of evidence, with data needing to converge in a triangulating fashion.”^1(p15)^ This design is described as a stand-alone research approach equivalent to grounded theory and can entail single and multiple cases.^1,2^ However, case study research should not be confused with single clinical case reports. “Case reports are familiar ways of sharing events of intervening with single patients with previously unreported features.”^3(p107)^ As a methodology, case study research encompasses substantially more complexity than a typical clinical case report.^1,3^

A particular characteristic of case study research is the use of various data sources, such as quantitative data originating from questionnaires as well as qualitative data emerging from interviews, observations, or documents. Therefore, a case study always draws on multiple sources of evidence, and the data must converge in a triangulating manner.^1^ When using multiple data sources, a case or cases can be examined more convincingly and accurately, compensating for the weaknesses of the respective data sources.^1^ Another characteristic is the interaction of various perspectives. This involves comparing or contrasting perspectives of people with different points of view, eg, patients, staff, or leaders.^4^ Through triangulation, case studies contribute to the completeness of the research on complex topics, such as role implementation in clinical practice.^1,5^ Triangulation involves a combination of researchers from various disciplines, of theories, of methods, and/or of data sources. By creating connections between these sources (ie, investigator, theories, methods, data sources, and/or data analysis), a new understanding of the phenomenon under study can be obtained.^6,7^

This scoping review focuses on methodologic and data-analysis triangulation because concrete procedures are missing, eg, in reporting guidelines. Methodologic triangulation has been called methods, mixed methods, or multimethods.^6^ It can encompass within-method triangulation and between/across-method triangulation.^7^ “Researchers using within-method triangulation use at least 2 data-collection procedures from the same design approach.”^6(p254)^ Within-method triangulation is either qualitative or quantitative but not both. Therefore, within-method triangulation can also be considered data source triangulation.^8^ In contrast, “researchers using between/across-method triangulation employ both qualitative and quantitative data-collection methods in the same study.”^6(p254)^ Hence, methodologic approaches are combined as well as various data sources. For this scoping review, the term “methodologic triangulation” is maintained to denote between/across-method triangulation. “Data-analysis triangulation is the combination of 2 or more methods of analyzing data.”^6(p254)^

Although much has been published on case studies, there is little consensus on the quality of the various data sources, the most appropriate methods, or the procedures for conducting methodologic and data-analysis triangulation.^5^ According to the EQUATOR (Enhancing the QUAlity and Transparency Of health Research) clearinghouse for reporting guidelines, one standard exists for organizational case studies.^9^ Organizational case studies provide insights into organizational change in health care services.^9^ Rodgers et al^9^ pointed out that, although high-quality studies are being funded and published, they are sometimes poorly articulated and methodologically inadequate. In the reporting checklist by Rodgers et al,^9^ a description of the data collection is included, but reporting directions on methodologic and data-analysis triangulation are missing. Therefore, the purpose of this study was to examine the process of methodologic and data-analysis triangulation in case studies. Accordingly, we conducted a scoping review to elicit descriptions of and directions for triangulation methods and analysis, drawing on case studies of nurse practitioners (NPs) in primary health care as an example. Case studies are recommended to evaluate the implementation of new roles in (primary) health care, such as that of NPs.^1,5^ Case studies on new role implementation can generate a unique and in-depth understanding of specific roles (individual), teams (smaller groups), family practices or similar institutions (organization), and social and political processes in health care systems.^1,10^ The integration of NPs into health care systems is at different stages of progress around the world.^11^ Therefore, studies are needed to evaluate this process.

## Methods

### Design

The methodological framework by Arksey and O’Malley^12^ guided this scoping review. We examined the current scientific literature on the use of methodologic and data-analysis triangulation in case studies on NPs in primary health care. The review process included the following stages: (1) establishing the research question; (2) identifying relevant studies; (3) selecting the studies for inclusion; (4) charting the data; (5) collating, summarizing, and reporting the results; and (6) consulting experts in the field.^12^ Stage 6 was not performed due to a lack of financial resources. The reporting of the review followed the PRISMA-ScR (Preferred Reporting Items for Systematic Reviews and Meta-Analyses extension for Scoping Review) guideline by Tricco et al^13^ (guidelines for reporting systematic reviews and meta-analyses [Supplementary Table A]). Scoping reviews are not eligible for registration in PROSPERO.

### Stage 1: Establishing the Research Question

The aim of this scoping review was to examine the process of triangulating methods and analysis in case studies on NPs in primary health care to improve the reporting. We sought to answer the following question: How have methodologic and data-analysis triangulation been conducted in case studies on NPs in primary health care? To answer the research question, we examined the following elements of the selected studies: the research question, the study design, the case definition, the selected data sources, and the methodologic and data-analysis triangulation.

### Stage 2: Identifying Relevant Studies

A systematic database search was performed in the MEDLINE (via PubMed) and CINAHL (via EBSCO) databases between July and September 2020 to identify relevant articles. The following terms were used as keyword search strategies: (“Advanced Practice Nursing” OR “nurse practitioners”) AND (“primary health care” OR “Primary Care Nursing”) AND (“case study” OR “case studies”). Searches were limited to English- and German-language articles. Hand searches were conducted in the journals *Nursing Inquiry*, *BMJ Open*, and *BioMed Central* (*BMC*). We also screened the reference lists of the studies included. The database search was updated in August 2023. The complete search strategy for all the databases is presented in Supplementary Table B.

### Stage 3: Selecting the Studies

#### Inclusion and exclusion criteria

We used the inclusion and exclusion criteria reported in Table 1. We included studies of NPs who had at least a master’s degree in nursing according to the definition of the International Council of Nurses.^14^ This scoping review considered studies that were conducted in primary health care practices in rural, urban, and suburban regions. We excluded reviews and study protocols in which no data collection had occurred. Articles were included without limitations on the time period or country of origin.

**Table 1.** Inclusion and Exclusion Criteria.

Criteria	Inclusion	Exclusion
Population	- NPs with a master’s degree in nursing or higher^14^	- Nurses with a bachelor’s degree in nursing or lower - Pre-registration nursing students - No definition of master’s degree in nursing described in the publication
Interest	- Description/definition of a case study design^1^ - Two or more data sources^1^	- Reviews - Study protocols - Summaries/comments/discussions
Context	- Primary health care - Family practices and home visits (including adult practices, internal medicine practices, community health centers)	- Nursing homes, hospital, hospice

#### Screening process

After the search, we collated and uploaded all the identified records into EndNote v.X8 (Clarivate Analytics, Philadelphia, Pennsylvania) and removed any duplicates. Two independent reviewers (MCS and SA) screened the titles and abstracts for assessment in line with the inclusion criteria. They retrieved and assessed the full texts of the selected studies while applying the inclusion criteria. Any disagreements about the eligibility of studies were resolved by discussion or, if no consensus could be reached, by involving experienced researchers (MZ-S and RP).

### Stages 4 and 5: Charting the Data and Collating, Summarizing, and Reporting the Results

The first reviewer (MCS) extracted data from the selected publications. For this purpose, an extraction tool developed by the authors was used. This tool comprised the following criteria: author(s), year of publication, country, research question, design, case definition, data sources, and methodologic and data-analysis triangulation. First, we extracted and summarized information about the case study design. Second, we narratively summarized the way in which the data and methodological triangulation were described. Finally, we summarized the information on within-case or cross-case analysis. This process was performed using Microsoft Excel. One reviewer (MCS) extracted data, whereas another reviewer (SA) cross-checked the data extraction, making suggestions for additions or edits. Any disagreements between the reviewers were resolved through discussion.

## Results

**Figure 1.**
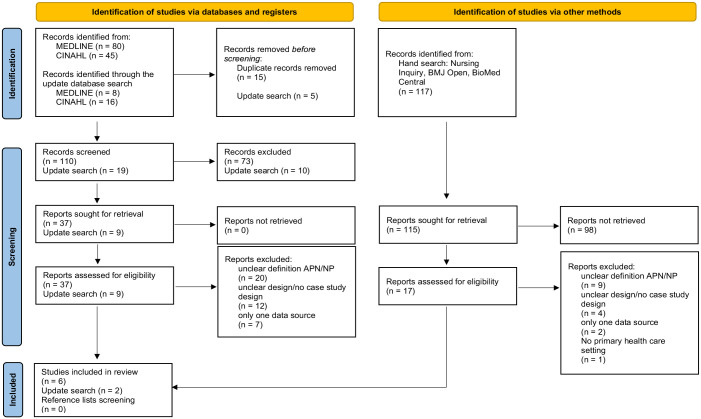
PRISMA flow diagram.

A total of 149 records were identified in 2 databases. We removed 20 duplicates and screened 129 reports by title and abstract. A total of 46 reports were assessed for eligibility. Through hand searches, we identified 117 additional records. Of these, we excluded 98 reports after title and abstract screening. A total of 17 reports were assessed for eligibility. From the 2 databases and the hand search, 63 reports were assessed for eligibility. Ultimately, we included 8 articles for data extraction. No further articles were included after the reference list screening of the included studies. A PRISMA flow diagram of the study selection and inclusion process is presented in Figure 1. As shown in Tables 2 and 3, the articles included in this scoping review were published between 2010 and 2022 in Canada (n = 3), the United States (n = 2), Australia (n = 2), and Scotland (n = 1).

**Table 2.** Characteristics of Articles Included.

Author	Contandriopoulos et al^15^	Flinter^16^	Hogan et al^17^	Hungerford et al^18^	O’Rourke^19^	Roots and MacDonald^20^	Schadewaldt et al^21^	Strachan et al^22^
Country	Canada	The United States	The United States	Australia	Canada	Canada	Australia	Scotland
How or why research question	No information on the research question	Several how or why research questions	What and how research question	No information on the research question	Several how or why research questions	No information on the research question	What research question	What and why research questions
Design and referenced author of methodological guidance	Six qualitative case studies Robert K. Yin	Multiple-case studies design Robert K. Yin	Multiple-case studies design Robert E. Stake	Case study design Robert K. Yin	Qualitative single-case study Robert K. Yin Robert E. Stake Sharan Merriam	Single-case study design Robert K. Yin Sharan Merriam	Multiple-case studies design Robert K. Yin Robert E. Stake	Multiple-case studies design
Case definition	Team of health professionals (Small group)	Nurse practitioners (Individuals)	Primary care practices (Organization)	Community-based NP model of practice (Organization)	NP-led practice (Organization)	Primary care practices (Organization)	No information on case definition	Health board (Organization)

**Table 3.** Overview of Within-Method, Between/Across-Method, and Data-Analysis Triangulation.

Author	Contandriopoulos et al^15^	Flinter^16^	Hogan et al^17^	Hungerford et al^18^	O’Rourke^19^	Roots and MacDonald^20^	Schadewaldt et al^21^	Strachan et al^22^
Within-method triangulation^a^ (using within-method triangulation use at least 2 data-collection procedures from the same design approach)								
*Qualitative approach*:								
Interviews	X		x	x	x			x
Observations			x	x				
Public documents	x				x			x
Electronic health records			x					
Between/across-method (using both qualitative and quantitative data-collection procedures in the same study)								
*Mixed-methods*:								
*Qualitative approach*:								
Interviews		x				x	x	
Observations						x	x	
Public documents						x	x	
Electronic health records		x						
*Quantitative approach*:								
Self-assessment		x						
Service records						x		
Questionnaires							x	
Data-analysis triangulation (combination of 2 or more methods of analyzing data)								
*Mixed-methods*:								
*Qualitative methods of analyzing*:								
Deductive		x				x	x	
Inductive						x	x	
Thematic						x	x	
Content								
*Quantitative methods of analyzing*:								
Descriptive analysis		x				x	x	
*Qualitative approach*:								
*Qualitative methods of analyzing*:								
Deductive	x			x	x			x
Inductive					x			x
Thematic								x
Content					x			

a Studies describing a quantitative approach and the triangulation of 2 or more quantitative data-collection procedures could not be included in this scoping review.

### Research Question, Case Definition, and Case Study Design

The following sections describe the research question, case definition, and case study design. Case studies are most appropriate when asking “how” or “why” questions.^1^ According to Yin,^1^ how and why questions are explanatory and lead to the use of case studies, histories, and experiments as the preferred research methods. In 1 study from Canada, eg, the following research question was presented: “How and why did stakeholders participate in the system change process that led to the introduction of the first nurse practitioner-led Clinic in Ontario?”^(p7)^^19^ Once the research question has been formulated, the case should be defined and, subsequently, the case study design chosen.^1^ In typical case studies with mixed methods, the 2 types of data are gathered concurrently in a convergent design and the results merged to examine a case and/or compare multiple cases.^10^

#### Research question

“How” or “why” questions were found in 4 studies.^16,17,19,22^ Two studies additionally asked “what” questions. Three studies described an exploratory approach, and 1 study presented an explanatory approach. Of these 4 studies, 3 studies chose a qualitative approach^17,19,22^ and 1 opted for mixed methods with a convergent design.^16^

In the remaining studies, either the research questions were not clearly stated or no “how” or “why” questions were formulated. For example, “what” questions were found in 1 study.^21^ No information was provided on exploratory, descriptive, and explanatory approaches. Schadewaldt et al^21^ chose mixed methods with a convergent design.

#### Case definition and case study design

A total of 5 studies defined the case as an organizational unit.^17,18-20,22^ Of the 8 articles, 4 reported multiple-case studies.^16,17,22,23^ Another 2 publications involved single-case studies.^19,20^ Moreover, 2 publications did not state the case study design explicitly.

### Within-Method Triangulation

This section describes within-method triangulation, which involves employing at least 2 data-collection procedures within the same design approach.^6,7^ This can also be called data source triangulation.^8^ Next, we present the single data-collection procedures in detail. In 5 studies, information on within-method triangulation was found.^15,17-19,22^ Studies describing a quantitative approach and the triangulation of 2 or more quantitative data-collection procedures could not be included in this scoping review.

#### Qualitative approach

Five studies used qualitative data-collection procedures. Two studies combined face-to-face interviews and documents.^15,19^ One study mixed in-depth interviews with observations,^18^ and 1 study combined face-to-face interviews and documentation.^22^ One study contained face-to-face interviews, observations, and documentation.^17^ The combination of different qualitative data-collection procedures was used to present the case context in an authentic and complex way, to elicit the perspectives of the participants, and to obtain a holistic description and explanation of the cases under study.

#### Interviews

All 5 studies used qualitative interviews as the primary data-collection procedure.^15,17-19,22^ Face-to-face, in-depth, and semi-structured interviews were conducted. The topics covered in the interviews included processes in the introduction of new care services and experiences of barriers and facilitators to collaborative work in general practices. Two studies did not specify the type of interviews conducted and did not report sample questions.^15,18^

#### Observations

In 2 studies, qualitative observations were carried out.^17,18^ During the observations, the physical design of the clinical patients’ rooms and office spaces was examined.^17^ Hungerford et al^18^ did not explain what information was collected during the observations. In both studies, the type of observation was not specified. Observations were generally recorded as field notes.

#### Public documents

In 3 studies, various qualitative public documents were studied.^15,19,22^ These documents included role description, education curriculum, governance frameworks, websites, and newspapers with information about the implementation of the role and general practice. Only 1 study failed to specify the type of document and the collected data.^15^

#### Electronic health records

In 1 study, qualitative documentation was investigated.^17^ This included a review of dashboards (eg, provider productivity reports or provider quality dashboards in the electronic health record) and quality performance reports (eg, practice-wide or co-management team-wide performance reports).

### Between/Across-Method Triangulation

This section describes the between/across methods, which involve employing both qualitative and quantitative data-collection procedures in the same study.^6,7^ This procedure can also be denoted “methodologic triangulation.”^8^ Subsequently, we present the individual data-collection procedures. In 3 studies, information on between/across triangulation was found.^16,20,21^

#### Mixed methods

Three studies used qualitative and quantitative data-collection procedures. One study combined face-to-face interviews, documentation, and self-assessments.^16^ One study employed semi-structured interviews, direct observation, documents, and service records,^20^ and another study combined face-to-face interviews, non-participant observation, documents, and questionnaires.^23^

#### Interviews

All 3 studies used qualitative interviews as the primary data-collection procedure.^16,20,23^ Face-to-face and semi-structured interviews were conducted. In the interviews, data were collected on the introduction of new care services and experiences of barriers to and facilitators of collaborative work in general practices.

#### Observation

In 2 studies, direct and non-participant qualitative observations were conducted.^20,23^ During the observations, the interaction between health professionals or the organization and the clinical context was observed. Observations were generally recorded as field notes.

#### Public documents

In 2 studies, various qualitative public documents were examined.^20,23^ These documents included role description, newspapers, websites, and practice documents (eg, flyers). In the documents, information on the role implementation and role description of NPs was collected.

#### Individual journals

In 1 study, qualitative individual journals were studied.^16^ These included reflective journals from NPs, who performed the role in primary health care.

#### Service records

Only 1 study involved quantitative service records.^20^ These service records were obtained from the primary care practices and the respective health authorities. They were collected before and after the implementation of an NP role to identify changes in patients’ access to health care, the volume of patients served, and patients’ use of acute care services.

### Questionnaires/Assessment

In 2 studies, quantitative questionnaires were used to gather information about the teams’ satisfaction with collaboration.^16,21^ In 1 study, 3 validated scales were used. The scales measured experience, satisfaction, and belief in the benefits of collaboration.^21^ Psychometric performance indicators of these scales were provided. However, the time points of data collection were not specified; similarly, whether the questionnaires were completed online or by hand was not mentioned. A competency self-assessment tool was used in another study.^16^ The assessment comprised 70 items and included topics such as health promotion, protection, disease prevention and treatment, the NP-patient relationship, the teaching-coaching function, the professional role, managing and negotiating health care delivery systems, monitoring and ensuring the quality of health care practice, and cultural competence. Psychometric performance indicators were provided. The assessment was completed online with 2 measurement time points (pre self-assessment and post self-assessment).

### Data-Analysis Triangulation

This section describes data-analysis triangulation, which involves the combination of 2 or more methods of analyzing data.^6^ Subsequently, we present within-case analysis and cross-case analysis.

#### Mixed-methods analysis

Three studies combined qualitative and quantitative methods of analysis.^16,20,21^ Two studies involved deductive and inductive qualitative analysis, and qualitative data were analyzed thematically.^20,21^ One used deductive qualitative analysis.^16^ The method of analysis was not specified in the studies. Quantitative data were analyzed using descriptive statistics in 3 studies.^16,20,23^ The descriptive statistics comprised the calculation of the mean, median, and frequencies.

#### Qualitative methods of analysis

Two studies combined deductive and inductive qualitative analysis,^19,22^ and 2 studies only used deductive qualitative analysis.^15,18^ Qualitative data were analyzed thematically in 1 study,^22^ and data were treated with content analysis in the other.^19^ The method of analysis was not specified in the 2 studies.

#### Within-case analysis

In 7 studies, a within-case analysis was performed.^15-20,22^ Six studies used qualitative data for the within-case analysis, and 1 study employed qualitative and quantitative data. Data were analyzed separately, consecutively, or in parallel. The themes generated from qualitative data were compared and then summarized. The individual cases were presented mostly as a narrative description. Quantitative data were integrated into the qualitative description with tables and graphs. Qualitative and quantitative data were also presented as a narrative description.

#### Cross-case analyses

Of the multiple-case studies, 5 carried out cross-case analyses. ^15-17,20,22^ Three studies described the cross-case analysis using qualitative data. Two studies reported a combination of qualitative and quantitative data for the cross-case analysis. In each multiple-case study, the individual cases were contrasted to identify the differences and similarities between the cases. One study did not specify whether a within-case or a cross-case analysis was conducted.^23^

#### Confirmation or contradiction of data

This section describes confirmation or contradiction through qualitative and quantitative data.^1,4^ Qualitative and quantitative data were reported separately, with little connection between them. As a result, the conclusions on neither the comparisons nor the contradictions could be clearly determined.

#### Confirmation or contradiction among qualitative data

In 3 studies, the consistency of the results of different types of qualitative data was highlighted.^16,19,21^ In particular, documentation and interviews or interviews and observations were contrasted:

- Confirmation between interviews and documentation: The data from these sources corroborated the existence of a common vision for an NP-led clinic.^19^
- Confirmation among interviews and observation: NPs experienced pressure to find and maintain their position within the existing system. Nurse practitioners and general practitioners performed complete episodes of care, each without collaborative interaction.^21^
- Contradiction among interviews and documentation: For example, interviewees mentioned that differentiating the scope of practice between NPs and physicians is difficult as there are too many areas of overlap. However, a clear description of the scope of practice for the 2 roles was provided.^21^

#### Confirmation through a combination of qualitative and quantitative data

Both types of data showed that NPs and general practitioners wanted to have more time in common to discuss patient cases and engage in personal exchanges.^21^ In addition, the qualitative and quantitative data confirmed the individual progression of NPs from less competent to more competent.^16^ One study pointed out that qualitative and quantitative data obtained similar results for the cases.^20^ For example, integrating NPs improved patient access by increasing appointment availability.

#### Contradiction through a combination of qualitative and quantitative data

Although questionnaire results indicated that NPs and general practitioners experienced high levels of collaboration and satisfaction with the collaborative relationship, the qualitative results drew a more ambivalent picture of NPs’ and general practitioners’ experiences with collaboration.^21^

## Discussion

### Research Question and Design

The studies included in this scoping review evidenced various research questions. The recommended formats (ie, how or why questions) were not applied consistently. Therefore, no case study design should be applied because the research question is the major guide for determining the research design.^2^ Furthermore, case definitions and designs were applied variably. The lack of standardization is reflected in differences in the reporting of these case studies. Generally, case study research is viewed as allowing much more freedom and flexibility.^5,24^ However, this flexibility and the lack of uniform specifications lead to confusion.

### Methodologic Triangulation

Methodologic triangulation, as described in the literature, can be somewhat confusing as it can refer to either data-collection methods or research designs.^6,8^ For example, methodologic triangulation can allude to qualitative and quantitative methods, indicating a paradigmatic connection. Methodologic triangulation can also point to qualitative and quantitative data-collection methods, analysis, and interpretation without specific philosophical stances.^6,8^ Regarding “data-collection methods with no philosophical stances,” we would recommend using the wording “data source triangulation” instead. Thus, the demarcation between the method and the data-collection procedures will be clearer.

### Within-Method and Between/Across-Method Triangulation

Yin^1^ advocated the use of multiple sources of evidence so that a case or cases can be investigated more comprehensively and accurately. Most studies included multiple data-collection procedures. Five studies employed a variety of qualitative data-collection procedures, and 3 studies used qualitative and quantitative data-collection procedures (mixed methods). In contrast, no study contained 2 or more quantitative data-collection procedures. In particular, quantitative data-collection procedures—such as validated, reliable questionnaires, scales, or assessments—were not used exhaustively. The prerequisites for using multiple data-collection procedures are availability, the knowledge and skill of the researcher, and sufficient financial funds.^1^ To meet these prerequisites, research teams consisting of members with different levels of training and experience are necessary. Multidisciplinary research teams need to be aware of the strengths and weaknesses of different data sources and collection procedures.^1^

### Data-Analysis Triangulation

#### Qualitative methods of analysis and results

When using multiple data sources and analysis methods, it is necessary to present the results in a coherent manner. Although the importance of multiple data sources and analysis has been emphasized,^1,5^ the description of triangulation has tended to be brief. Thus, traceability of the research process is not always ensured. The sparse description of the data-analysis triangulation procedure may be due to the limited number of words in publications or the complexity involved in merging the different data sources.

Only a few concrete recommendations regarding the operationalization of the data-analysis triangulation with the qualitative data process were found.^25^ A total of 3 approaches have been proposed^25^: (1) the intuitive approach, in which researchers intuitively connect information from different data sources; (2) the procedural approach, in which each comparative or contrasting step in triangulation is documented to ensure transparency and replicability; and (3) the intersubjective approach, which necessitates a group of researchers agreeing on the steps in the triangulation process. For each case study, one of these 3 approaches needs to be selected, carefully carried out, and documented. Thus, in-depth examination of the data can take place. Farmer et al^25^ concluded that most researchers take the intuitive approach; therefore, triangulation is not clearly articulated. This trend is also evident in our scoping review.

#### Mixed-methods analysis and results

Few studies in this scoping review used a combination of qualitative and quantitative analysis. However, creating a comprehensive stand-alone picture of a case from both qualitative and quantitative methods is challenging. Findings derived from different data types may not automatically coalesce into a coherent whole.^4^ O’Cathain et al^26^ described 3 techniques for combining the results of qualitative and quantitative methods: (1) developing a triangulation protocol; (2) following a thread by selecting a theme from 1 component and following it across the other components; and (3) developing a mixed-methods matrix.

The most detailed description of the conducting of triangulation is the triangulation protocol. The triangulation protocol takes place at the interpretation stage of the research process.^26^ This protocol was developed for multiple qualitative data but can also be applied to a combination of qualitative and quantitative data.^25,26^ It is possible to determine agreement, partial agreement, “silence,” or dissonance between the results of qualitative and quantitative data. The protocol is intended to bring together the various themes from the qualitative and quantitative results and identify overarching meta-themes.^25,26^

The “following a thread” technique is used in the analysis stage of the research process. To begin, each data source is analyzed to identify the most important themes that need further investigation. Subsequently, the research team selects 1 theme from 1 data source and follows it up in the other data source, thereby creating a thread. The individual steps of this technique are not specified.^26,27^

A mixed-methods matrix is used at the end of the analysis.^26^ All the data collected on a defined case are examined together in 1 large matrix, paying attention to cases rather than variables or themes. In a mixed-methods matrix (eg, a table), the rows represent the cases for which both qualitative and quantitative data exist. The columns show the findings for each case. This technique allows the research team to look for congruency, surprises, and paradoxes among the findings as well as patterns across multiple cases. In our review, we identified only one of these 3 approaches in the study by Roots and MacDonald.^20^ These authors mentioned that a causal network analysis was performed using a matrix. However, no further details were given, and reference was made to a later publication. We could not find this publication.

### Case Studies in Nursing Research and Recommendations

**Figure 2.**
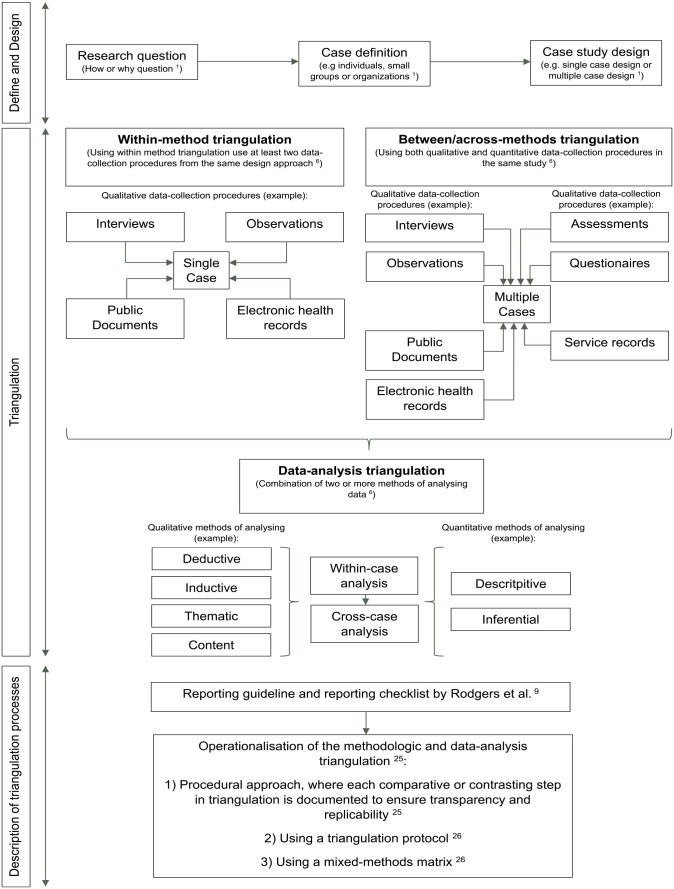
Schematic representation of methodologic and data-analysis triangulation in case studies (own work).

Because it focused on the implementation of NPs in primary health care, the setting of this scoping review was narrow. However, triangulation is essential for research in this area. This type of research was found to provide a good basis for understanding methodologic and data-analysis triangulation. Despite the lack of traceability in the description of the data and methodological triangulation, we believe that case studies are an appropriate design for exploring new nursing roles in existing health care systems. This is evidenced by the fact that case study research is widely used in many social science disciplines as well as in professional practice.^1^ To strengthen this research method and increase the traceability in the research process, we recommend using the reporting guideline and reporting checklist by Rodgers et al.^9^ This reporting checklist needs to be complemented with methodologic and data-analysis triangulation. A procedural approach needs to be followed in which each comparative step of the triangulation is documented.^25^ A triangulation protocol or a mixed-methods matrix can be used for this purpose.^26^ If there is a word limit in a publication, the triangulation protocol or mixed-methods matrix needs to be identified. A schematic representation of methodologic and data-analysis triangulation in case studies can be found in Figure 2.

### Limitations

This study suffered from several limitations that must be acknowledged. Given the nature of scoping reviews, we did not analyze the evidence reported in the studies. However, 2 reviewers independently reviewed all the full-text reports with respect to the inclusion criteria. The focus on the primary care setting with NPs (master’s degree) was very narrow, and only a few studies qualified. Thus, possible important methodological aspects that would have contributed to answering the questions were omitted. Studies describing the triangulation of 2 or more quantitative data-collection procedures could not be included in this scoping review due to the inclusion and exclusion criteria.

## Conclusions

Given the various processes described for methodologic and data-analysis triangulation, we can conclude that triangulation in case studies is poorly standardized. Consequently, the traceability of the research process is not always given. Triangulation is complicated by the confusion of terminology. To advance case study research in nursing, we encourage authors to reflect critically on methodologic and data-analysis triangulation and use existing tools, such as the triangulation protocol or mixed-methods matrix and the reporting guideline checklist by Rodgers et al,^9^ to ensure more transparent reporting.

## Supplemental Material

sj-docx-1-wjn-10.1177_01939459241263011 – Supplemental material for Methodologic and Data-Analysis Triangulation in Case Studies: A Scoping Review Supplemental material, sj-docx-1-wjn-10.1177_01939459241263011 for Methodologic and Data-Analysis Triangulation in Case Studies: A Scoping Review by Margarithe Charlotte Schlunegger, Maya Zumstein-Shaha and Rebecca Palm in Western Journal of Nursing Research
